# Development and Genetic Characterization of Advanced Backcross Materials and An Introgression Line Population of *Solanum incanum* in a *S. melongena* Background

**DOI:** 10.3389/fpls.2017.01477

**Published:** 2017-08-30

**Authors:** Pietro Gramazio, Jaime Prohens, Mariola Plazas, Giulio Mangino, Francisco J. Herraiz, Santiago Vilanova

**Affiliations:** ^1^Instituto de Conservación y Mejora de la Agrodiversidad Valenciana, Universitat Politècnica de València Valencia, Spain; ^2^Instituto de Biología Molecular y Celular de Plantas, Consejo Superior de Investigaciones Científicas - Universitat Politècnica de València Valencia, Spain

**Keywords:** *Solanum melongena*, *Solanum incanum*, advanced backcrosses, introgression lines, drought tolerance, high-throughput genotyping

## Abstract

Advanced backcrosses (ABs) and introgression lines (ILs) of eggplant (*Solanum melongena*) can speed up genetics and genomics studies and breeding in this crop. We have developed the first full set of ABs and ILs in eggplant using *Solanum incanum*, a wild eggplant that has a relatively high tolerance to drought, as a donor parent. The development of these ABs and IL eggplant populations had a low efficiency in the early stages, because of the lack of molecular markers and genomic tools. However, this dramatically improved after performing genotyping-by-sequencing in the first round of selfing, followed by high-resolution-melting single nucleotide polymorphism genotyping in subsequent selection steps. A set of 73 selected ABs covered 99% of the *S. incanum* genome, while 25 fixed immortal ILs, each carrying a single introgressed fragment in homozygosis, altogether spanned 61.7% of the *S. incanum* genome. The introgressed size fragment in the ILs contained between 0.1 and 10.9% of the *S. incanum* genome, with a mean value of 4.3%. Sixty-eight candidate genes involved in drought tolerance were identified in the set of ILs. This first set of ABs and ILs of eggplant will be extremely useful for the genetic dissection of traits of interest for eggplant, and represents an elite material for introduction into the breeding pipelines for developing new eggplant cultivars adapted to the challenges posed by the climate-change scenario.

## Introduction

Eggplant (*Solanum melongena* L., 2n = 2x = 24) is a major vegetable crop of increasing importance during the last few years; while other major crops in the genus *Solanum* and in the family Solanaceae, like tomato, potato, pepper, tobacco, and petunia, have been widely studied from the genetic and genomic points of view, developments in eggplant genomics have lagged behind. In fact, the first draft of the eggplant genome was published only in 2014 (Hirakawa et al., 2014), while in the other major solanaceous crops high-quality genome sequences were available much earlier (Potato Genome Sequencing Consortium, 2011; Tomato Genome Consortium, 2012). While other eggplant genomic tools are slowly being published, like the first transcriptome (Yang et al., 2014), genetic and association maps (Barchi et al., 2012; Fukuoka et al., 2012; Gramazio et al., 2014; Portis et al., 2015) and quantitative trait locus (QTL) studies (Miyatake et al., 2016; Rinaldi et al., 2016; Toppino et al., 2016), there is a general lack of eggplant experimental populations, such as recombinant inbred lines (RILs), advanced backcrosses (ABs), introgression lines (ILs) or multiparent advanced generation intercross (MAGIC) populations.

However, although a great phenotypic diversity can be found in the common eggplant (Portis et al., 2015; Kaushik et al., 2016), its genetic base is quite narrow due to the genetic bottlenecks undergone during the domestication and subsequent crop evolution processes (Muñoz-Falcón et al., 2009; Meyer et al., 2012). This low genetic diversity could be a serious constraint for the development of new highly performing cultivars, which have to be able to contribute to increasing yield under the environmental changes resulting from a climate-change scenario (Tilman et al., 2011; Ray et al., 2013).

Although, many resistances and tolerances to biotic and abiotic stresses have been described in eggplant wild relatives (Rotino et al., 2014), just a few attempts have been made to transfer them to the cultivated eggplant (Toppino et al., 2008; Liu et al., 2015). Recent efforts in enhancing wild relatives for the adaptation of eggplant to climate change have resulted in the development of over 50 combinations of hybrids and 30 first backcross (BC1) generations (Kouassi et al., 2016; Plazas et al., 2016). These materials were obtained from six *S. melongena* parents and 14 wild species from different genepools, including the primary genepool (GP1, which includes relatives that can be easily crossed with the cultivated species to produce a highly fertile progeny), secondary genepool (GP2, which includes species that can be crossed with the cultivated species, although with some difficulties due to reproductive barriers, and that may give a low fertility progeny) and the tertiary genepool (GP3, which is made of phylogenetically distant species where pre- and post-zygotic barriers require specific breeding techniques, like embryo rescue, for hybridization and the progeny is often sterile).

The development of ABs and ILs in the eggplant genepool would make a major contribution to enhancing the use of crop wild relatives in eggplant breeding (Prohens et al., 2017). ABs consist of materials obtained through a backcross breeding scheme containing fixed or non-fixed single or multiple introgressions from a donor in the genetic background of a recurrent parent (Fulton et al., 1997). ABs represent powerful tools that allow more precise mapping of QTLs compared to balance populations (e.g., F2, BC1, RIL) and an elite material for breeding (Tanksley and Nelson, 1996). IL collections are a special type of AB population, consisting of fixed lines with single introgressed fragments from a donor parent (Eshed and Zamir, 1995; Tian et al., 2006). ILs are immortal populations making them more suitable for direct incorporation into breeding pipelines. The general aim for developing sets of ILs is to represent the whole genome of a donor parent, almost always an exotic or wild genotype, in the genetic background of a recipient cultivated parent through a set of lines carrying overlapping homozygous chromosome fragments of the former in the latter (Eshed and Zamir, 1994; Zamir, 2001; Eduardo et al., 2005). Several studies have demonstrated the higher efficiency of ILs and near isogenic lines (NILs) in QTL estimation than other segregating populations, such as F2, double haploid lines and RILs (Wehrhahn and Allard, 1965; Eshed and Zamir, 1995; Kearsey and Farquhar, 1998; Lebreton et al., 1998; Alonso-Blanco et al., 2006; Yin et al., 2016).

Although, the availability of IL populations in crop species is still limited, in the last two decades many have been developed, mainly in major cultivated crops such as maize, wheat, rice, barley, soybean, and melon (Koester et al., 1993; Concibido et al., 2003; Von Korff et al., 2004; Eduardo et al., 2005; Liu et al., 2006; Pestsova et al., 2006; Tian et al., 2006). The availability of a large number of IL populations in tomato has promoted and facilitated studies of the genetics of morphology (Chitwood et al., 2013), physiology (Ranjan et al., 2016), biotic resistance (Verlaan et al., 2013), abiotic tolerance (Albacete et al., 2015), chemical composition (Ikeda et al., 2017), enzyme activity (Caruso et al., 2016), metabolic profiling (Schauer et al., 2006), transcriptional profiles (Baxter et al., 2005), and many more traits.

In the case of eggplant, up until now no sets of ABs or ILs covering the whole genome of a relative have been obtained. In order to obtain the first set of introgression materials of this type in eggplant, we chose *Solanum incanum* L. as a donor parent. This wild species belongs to the secondary genepool of common eggplant (Syfert et al., 2016), and is naturally distributed in the desertic and dryland areas from western Pakistan, Afghanistan, and Iran across the Middle East and northern Africa (Ranil et al., 2015; Vorontsova and Knapp, 2016). *S. incanum* is reported as having considerable drought tolerance (Lester and Hasan, 1991; Daunay, 2008; Knapp et al., 2013), which has been confirmed under experimental conditions (Savarino, 2017). Drought tolerance is a trait of major interest for adaptation to climate change, where new drought-tolerant varieties are required (Dempewolf et al., 2014). In addition, *S. incanum* presents resistance to some fungal diseases, such as *Fusarium oxysporum* and *Phomopsis vexans* (Yamakawa and Mochizuki, 1979; Collonnier et al., 2001). Also, *S. incanum* exhibits a high content of bioactive phenolic compounds, mostly of chlorogenic acid type (Stommel and Whitaker, 2003; Ma et al., 2011; Prohens et al., 2013; Meyer et al., 2015). These favorable features led us to use *S. incanum* for the construction of an interspecific genetic map with *S. melongena*, which is linked to four eggplant and one tomato genetic maps (Gramazio et al., 2014). Furthermore, the transcriptome of *S. incanum* was sequenced to create a large set of molecular markers directly applicable in breeding programs and for a better understanding of the target genes involved in metabolic pathways relevant for eggplant breeding (Gramazio et al., 2016). Also, recently, the entire genome of *S. incanum* has been sequenced (Gramazio, unpublished data).

Here, we present the development of the first full set of ABs and ILs in the eggplant genetic background, from the first generations, where little genomic information was available and low–medium throughput genotyping techniques were used, to the last ones, where more markers were available and affordable high-throughput genotyping-by-sequencing (GBS) helped in the characterization of the ABs and in obtaining a set of ILs with introgressions containing a single fragment from the *S. incanum* donor.

## Materials and methods

### Plant material

**Figure 1:**
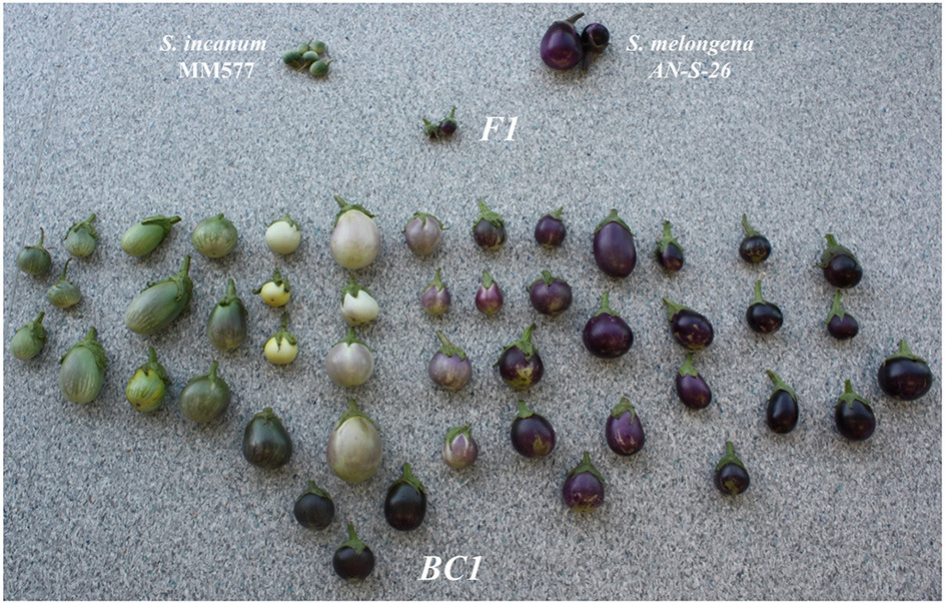
Parents (*Solanum incanum* MM577 and *S. melongena* AN-S-26) of the AB and IL sets, their hybrid (F1) and the segregating first backcross (BC1) generation to the recipient parent *S. melongena* AN-S-26.

The donor parent selected for the development of the set of ABs and ILs was *S. incanum* accession number MM577, collected in the wild in Israel and provided by the INRA Avignon (France) germplasm bank. MM577 plants are prickly, with no anthocyanin pigmentation and produce small green rounded fruits (Figure 1). This MM577 plant has been used in several genetic and genomic studies, as it has been characterized for morphological traits and phenolic acids content (Stommel and Whitaker, 2003), for androgenic competence through anther culture (Salas et al., 2011), as a rootstock for eggplant yield and its effects on fruit quality (Gisbert et al., 2011), for phylogeographic studies (Meyer et al., 2015), for breeding for adaptation to climate change (http://eggplantprebreeding.upv.es/), as a parental of an interspecific genetic map (Gramazio et al., 2014), for transcriptome sequencing (Gramazio et al., 2016) and for whole genome resequencing (Gramazio, unpublished data).

The recipient parent was *S. melongena* accession number AN-S-26, a Spanish local variety from Andalusia obtained from the Universitat Politècnica de València (UPV), Valencia, Spain, germplasm bank. The AN-S-26 plant is non-prickly, with anthocyanin pigmentation and large obovoid dark purple fruits (Figure 1). The morphological characterization and phenolics content of *S. incanum* MM577 and *S. melongena* AN-S-26 revealed important differences in plant and fruit morphology and phenolic concentration between them (Prohens et al., 2013). The genetic characterization of both parents with simple sequence repeat (SSR) and conserved ortholog set of genes II (COSII) markers also revealed a high level of genetic polymorphism betwee them (Gramazio et al., 2014). Both accessions numbersare maintained at the UPV germplasm genebank.

Seeds were germinated in Petri dishes and were subsequently transferred to seedling trays. These were kept in a climatic chamber under a photoperiod and temperature regime of 16 h light (25°C): 8 h dark (18°C). The plantlets were then transplanted to a pollinator-free glasshouse situated in the campus of the UPV, Valencia, Spain (GPS coordinates: latitude, 39° 28′ 55″ N; longitude, 0° 20′ 11″ W; 7 m above sea level). Plants were grown in 15 L pots filled with coconut fiber, irrigated and fertilized using a drip irrigation system, and pruned and trained with vertical strings. Phytosanitary treatments against spider mites and whiteflies were performed when necessary. Plants were hand pollinated and crosses were performed between the recurrent parent and the selected descendants of a family (hereafter referred to as progeny). *S. melongena* AN-S-26 was used mainly as the male parent, except for when the F1 and BC1 generations were obtained, where AN-S-26 was the female parent.

The hand pollinations were carried out as follows: the flowers of the female parent were emasculated before anthesis (i.e., before flower opening), then pollen was deposited on the stigma by gently rubbing it with a glass slide covered with the pollen of the male parent. Pollinated flowers were covered with a paper or mesh bag in order to avoid undesired cross-pollination, and tagged with information on the cross or selfing performed and the date.

### Breeding scheme

**Figure 2:**
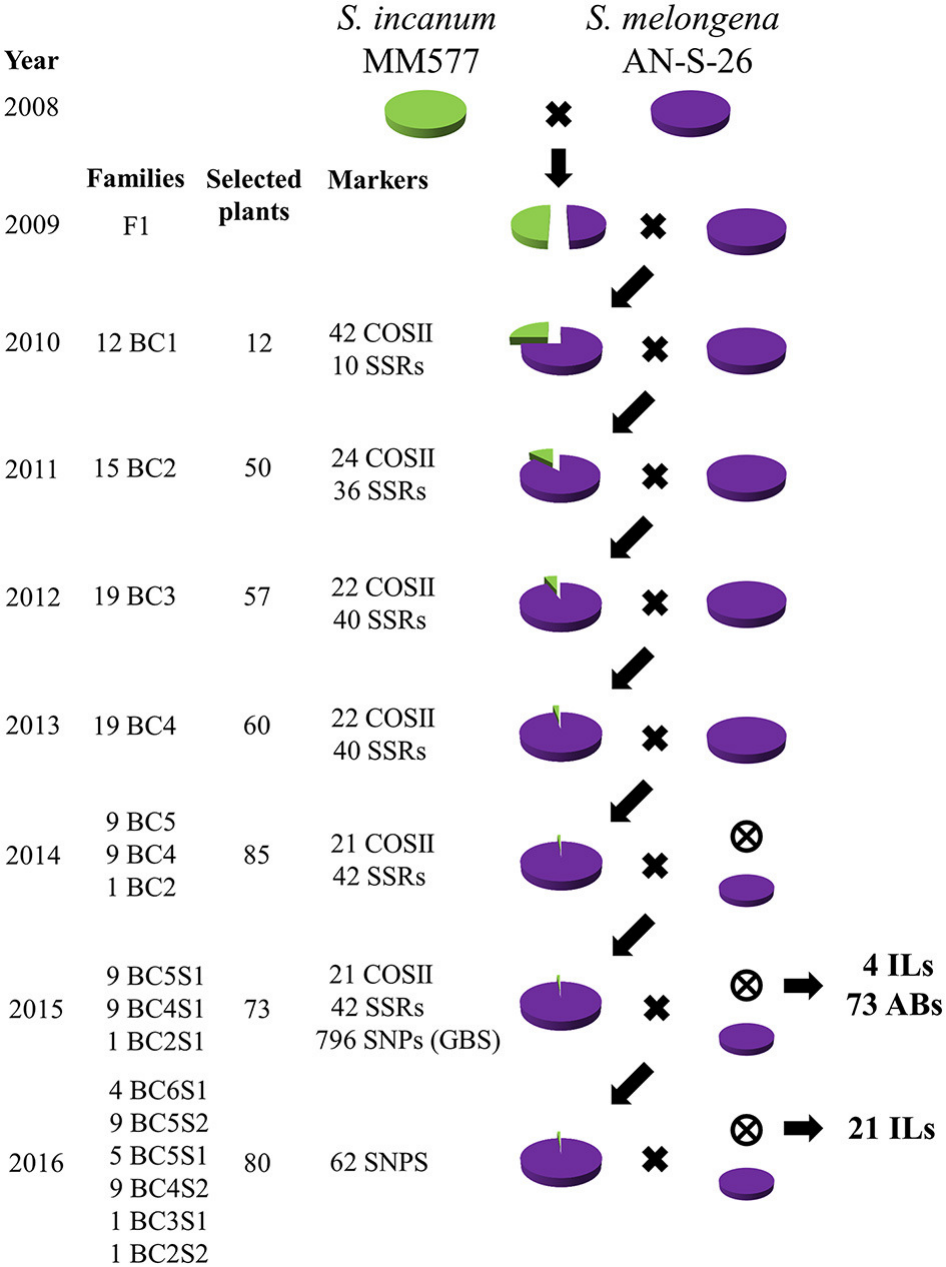
Breeding scheme used to develop the first set of eggplant ABs and ILs in the genetic background of *Solanum melongena* AN-S-26 (purple circle) by using *S. incanum* MM577 (green circle) as a donor. For each year (one generation per year), the number of families, selected plants, molecular markers assessed and type of cross are shown, the backcrosses with the recurrent parents are represented by an × and a purple circle, and the selfing rounds by an encircled ×.

The breeding scheme used to develop the ABs and ILs is depicted in Figure 2. The F1 hybrid was obtained in 2008 using the recipient parent as a female. The hybrid was completely fertile and in the following year was backcrossed to the AN-S-26 parent to generate the BC1 population.

The BC1 population, which consisted of 91 plants, was used to build the interspecific genetic map (Gramazio et al., 2014). A total of 12 selected BC1 plants (hereafter referred to as families) were backcrossed to generate the BC2 population. A subset of 50 selected BC2 plants, four to five per BC1 family, were grown in the greenhouse and backcrossed to obtain the BC3 population.

Twenty plants of each BC3 family were genotyped and three of each family were selected for the next round of backcross. Fifteen to twenty BC4 plants were genotyped at the seedling stage and 60 of them were selected for producing the next backcross generation. Some BC4 families did not set fruit; thus in 2014, in addition to the BC5 plants, some BC4 and BC2 plants, which had not given any progeny, were genotyped and selfed for a total of 85 plants.

After a preliminary manual genotyping of the progenies obtained, and driven by the recent publication of the first draft of the eggplant genome (Hirakawa et al., 2014), a high-throughput GBS was performed for the selected selfed genotypes. A total of 73 genotyped plants, including BC5S1, BC4S1, and BC2S1, were grown in the greenhouse and selfed to produce seeds in order to obtain the set of ABs and a first subset of ILs with single homozygous introgressions. In addition, the plants that presented two or more homozygous introgressions were backcrossed in order to obtain single homozygous introgressions.

A last round of selection for obtaining fixed ILs, including BC6S1, BC5S2, BC5S1, BC4S2, BC3S1, and BC2S2 families, was made with a new set of single nucleotide polymorphisms (SNPs) obtained by the GBS method based on the physical position. An additional 21 ILs with a single homozygous introgression, were identified. A total of 80 selected plants, around three to four per family, were grown in the greenhouse. Depending on the introgressions carried, they were selfed to produce seeds for the new ILs identified and to reduce the residual heterozygosity, or backcrossed to reduce the number of homozygous and off-target introgressions.

### DNA extraction

Total genomic DNA was isolated from young leaves of plantlets at the 3–4 true leaves stage according to the CTAB method (Doyle and Doyle, 1987), with slight modifications. The extracted DNA was dissolved in Milli-Q water and its quality was checked in a 0.8% agarose gel. DNA concentration was measured with a Qubit® 2.0 Fluorometer (Thermo Fisher Scientific, Waltham, USA) and adjusted to 30 ng/μL for PCR and high resolution melting (HRM) amplification (Wittwer et al., 2003) and to 100 ng/μL for GBS.

### Genotyping methods

At the same time as the ABs and ILs were developed, a genetic linkage map was built from a cross between the parents, and was anchored to four eggplant and one tomato genetic maps (Gramazio et al., 2014). This map comprised 42 COSII, 99 SSRs, 88 amplified fragment length polymorphisms (AFLPs), 9 cleaved amplified polymorphic sequences (CAPS), 4 SNPs, and one morphological polymorphic markers, and encompassed 1,085 cM distributed in 12 linkage groups. A subset of these markers was used to assist in the selection of the plants during the development of the ABs and ILs.

Forty-two universal primers of COSII markers (Wu et al., 2006), which were previously mapped in an interspecific genetic map between *S. linnaeanum* and *S. melongena* (Wu et al., 2009), and 10 SSRs, developed by Nunome et al. (2009), which corresponded to the first set of markers mapped, were used to genotype the 91 BC1 plants and to select the 12 BC1 used to generate the BC2 population. The 12 plants selected presented all the markers from the donor for the target chromosome, each one different from the other 11 BC1 plants. The 240 BC2 progeny were genotyped at the seedling stage with 60 markers, 24 COSII and 36 SSRs, some of which were developed by our group (Vilanova et al., 2012). The details of the marker types, polymorphism and segregation distortion were reported in Gramazio et al. (2016). The new SSRs substituted for some of the COSII markers for reaching new genetic positions uncovered by the latter. For the BC2 population, not the whole background of each plant was screened, but just the target chromosome of each family. So, for example, the BC2 plants derived from the BC1 plant selected for chromosome 1 were genotyped only for chromosome 1 and not for chromosomes 2–12.

This strategy was employed to reduce the time and resources required to assess all the markers in all the chromosomes, and to perform genotyping at the seedling stage in order to grow only the selected plants to the reproductive stage. In fact, the approach was to develop, as a first step and as long as possible, a set of chromosome substitution lines in order to reduce the number of plants to be genotyped, grown, and backcrossed. The set of markers used for genotyping the BC3, BC4, BC5, and BC5S1 generations was practically the same, except for a few changes where SSRs replaced some COSII markers.

The selected plants from the first round of selfing were also genotyped with a GBS platform. A total of 796 SNPs was selected to improve the estimation of recombination breakpoints and to detect non-target introgressions that were dragged during the ABs and ILs development. From the whole collection of SNPs detected by GBS, 62 of them were selected, from four to eight markers per chromosome, and adapted for HRM analysis to genotype the plants involved in the second round of selfing. The number of SNPs assessed for each progeny was variable, depending on the size and the type (target or not-target) of the introgressions. The size and the position of the introgressions in megabases (Mb) and the percentage of coverage were calculated according to the physical position of the new eggplant genome assembly developed by the Italian Eggplant Genome Sequencing Consortium (Barchi et al., 2016). Primers pairs were designed using the Primer3 tool (v. 0.4.0, http://bioinfo.ut.ee/primer3-0.4.0/primer3/) with a mean amplicon range of 80–120 bp.

### COSII marker analysis

COSII primer pairs were amplified by PCR in a 12 μL reaction volume comprising 7.21 μL water, 1.2 μL 1 × PCR buffer, 0.6 μL MgCl_2_ (50 mM), 0.24 μL dNTPs (10 mM), 0.3 μL of each primer (10 μM), 0.15 μL *Taq* DNA Polymerase (5 U/μL), and 2 μL DNA template (20 ng/μL), under the following cycling conditions: denaturation at 95°C for 3 min; followed by 30 cycles of 30 s at 95°C, 30 s at 65°C, and of 30 s at 72°C; with a final extension at 72°C for 5 min. Then, restriction enzymes were used to cut in the polymorphic regions and the different size bands were visualized in 2–3% agarose gel.

### SSR marker analysis

The amplification of SSRs was performed by PCR in a final volume of 12 μL: 7.21 μL water, 1.2 μL 1 × PCR buffer, 0.6 μL MgCl_2_ (50 mM), 0.24 μL dNTPs (10 mM), 0.3 μL reverse primer (10 μM), 0.06 μL forward primer with M13 tail (10 μM), 0.24 μL fluorochrome (FAM, VIC, NED, and PET) (10 μM), 0.15 μL *Taq* DNA Polymerase (5 U/μL), and 2 μL DNA template (20 ng/μL). The PCR amplification program consisted of: a first step of denaturation at 95°C for 3 min; followed by 30 cycles of 30 s at 95°C, 30 s at 65°C, and of 30 s at 72°C; and a last step of extension at 72°C for 5 min. Subsequently, the PCR products were diluted in formamide and analyzed with an automated ABI PRISM 3100-*Avant* DNA sequencer, with a GeneScan 600LIZ (Applied Biosystems, California, USA) size standard. The fragments were analyzed using GeneScan software (Applied Biosystems) to obtain the electropherograms and polymorphisms were analyzed with Genotyper DNA Fragment Analysis software (Applied Biosystems).

### SNP marker analysis

SNPs were validated in a LightCycler 480 Real-Time PCR (Roche, Basel, Switzerland). The reactions were performed in a 10 μL volume comprising 5 μL Master Mix (2×), 0.8 μL MgCl_2_ (25 mM), 0.25 μL each primer, 1.7 μL water, and 2 μL DNA (30 ng/μL) with the following touchdown PCR program: denaturation at 95°C for 10 min; followed by 55 cycles of 10 s at 95°C, 15 s at 65°C (decreasing 1°C each cycle until 55°C) and 15 s at 72°C; and a final melting step of 1 min at 95°C, 1 min at 40°C, and 1 s at 60°C with the temperature being raised by 0.02°C/s until 95°C was reached.

### GBS analysis

GBS was performed in 83 plants comprising 73 pre-selected plants from different generations (BC5S1, BC4S1, and BC2S1), 2 samples of each parent, 2 additional samples of doubled haploids of the recurrent parent and 2 samples of the hybrid. After DNA extraction and dilution, the samples were sent to the Cornell University genomic facilities in August 2015 for library preparation (a PstI enzyme was used) and sequencing. The raw data were demultiplexed and the Illumina barcode removed using the software GBS Barcode Splitter (https://sourceforge.net/projects/gbsbarcode/). Subsequently, the sequences were filtered at a 25 Phred quality score and the Illumina adapters removed with the Fastq-mcf program (Aronesty, 2013). A quality control before and after the trimming was performed with the FastQC tool (Andrews, 2010) and general statistics were obtained using Fastq-stats (Aronesty, 2013). Then, the clean reads were mapped against the reference published genome (Hirakawa et al., 2014) and against another eggplant genome with improved quality, kindly provided by the Italian Eggplant Genome Sequencing Consortium (Barchi et al., 2016). Due to the better assembly of the latter (12 pseudomolecules vs. 33,873 scaffolds of the former), we decided to use it for the further analysis. The mapping was performed using BWA-MEM software (Li, 2013) with default settings and the SNP calling using the FreeBayes program (Garrison and Marth, 2012), ignoring those variants that presented less coverage than 15, a mapping quality of 20 or a base quality of 20. In order to select the most reliable SNPs for genotyping the plants with HRM, different filters from the Seq Crumbs tools (https://bioinf.comav.upv.es/seq_crumbs/) were applied to the VCF file for maximizing the polymorphism validation. The filters CS60 and CL60 identified polymorphisms that were at a distance of < 60 nucleotides to another polymorphism or to the alignment edge, respectively. The filter HV0.05 determined whether the region had more than 5 polymorphisms per 100 bases, while the filter VKS discriminated INDELs from SNPs. GBS raw data were deposited in the NCBI Sequence Read Archive (http://www.ncbi.nlm.nih.gov/sra/) repository under the accession number SAMN07249022.

### Positioning candidate genes for drought tolerance

Candidate genes involved in pathways strictly related to drought tolerance were gathered from the current scientific literature (Bolger et al., 2014; Zhang N. et al., 2014; Krannich et al., 2015; Landi et al., 2016). Orthologous genes of these candidates were searched for in tomato (*Solanum lycopersicum*) and potato (*Solanum tuberosum*) through EnsemblPlants (http://plants.ensembl.org/index.html/) and NCBI (National Center for Biotechnology Information; https://www.ncbi.nlm.nih.gov/) databases, and the corresponding Fasta sequences were retrieved. Subsequently, a Blast search (with a cut-off value of 1e-60) was performed with the Fasta sequences to position the candidate genes on the eggplant genome (Barchi et al., 2016) and to assign them to the corresponding ILs.

## Results

### Development of the ABs and ILs

A total of 91 BC1 plants were obtained by crossing the F1 hybrid *S. melongena* AN-S-26 × *S. incanum* MM577 to the recurrent parent (AN-S-26), generating a highly diverse population in plant morphology and in fruit color, shape, size, and prickles (Figure 1). Based on the information of the 52 molecular markers assessed, 12 BC1 plants were selected. Each of them was heterozygous for all the scored markers of the target chromosome and had a reduced percentage of donor parent genetic background in the rest of their chromosomes. The BC2 progeny, obtained from the 12 BC1 plants, were genotyped with a mean of four to five molecular markers (24 COSII and 36 SSRs). This allowed selection of 15 BC2 families, which were taken to the greenhouse in order to obtain the BC3 population. For chromosomes 5, 7, and 8, no individual plants displayed a chromosome with all scored markers completely heterozygous; thus, two complementary individuals for each of them were selected to cover all the markers for the target chromosomes. As in the previous year (2011), the selected plants were transplanted to the greenhouse at the end of October with the aim of obtaining two cycles of backcross per year (winter–spring and summer–fall). For several reasons, including seed dormancy, slow growth, poor flowering with low-quantity pollen during winter and parthenocarpy, just one cycle per year was achievable.

The BC3 progeny were genotyped with 22 COSII and 38 SSRs. Four chromosomes (1, 2, 4, and 10) were not completely heterozygous for the markers used in any of the selected plants; hence, the number of progeny rose to nineteen. The genotyping of the BC4 plants (22 COSII and 42 SSRs) allowed selection of the progeny for the last round of backcross. Unfortunately, nine BC4 families (two from chromosome 1, two from chromosome 2, one from chromosome 6, two from chromosome 7, one from chromosome 10, and one from chromosome 11) did not produce fruit due to poor fruit flowering and setting. Thus, in order to avoid loss of these introgressions, new BC4 families for those from which we could not obtain seeds were genotyped again, together with the progenies of the BC5 families. Moreover, for chromosome 6, the few remaining seeds of the BC4 and BC3 generations did not germinate and the BC2 progeny were used to continue the process.

The first round of selfing was performed for nine BC5, nine BC4, and one BC2 families. In addition, the BC4 and BC2 families were backcrossed for a backup, due to the fact that the BC2 and most of the BC4 generations that were re-genotyped had few remaining seeds. The progenies obtained from selfing were manually genotyped with 24 COSII and 44 SSRs. Subsequently, 73 plants that were pre-selected from the BC5S1, BC4S1, and BC2S1 families were additionally sequenced through GBS. The selfed offspring of these 73 plants constituted the ABs set.

The genotyping by GBS allowed a very accurate detection of introgression length, precisely defining the recombination breakpoints and the double recombination events undetected due to the low–medium density molecular marker assisted selection used in the previous generations. The sequencing of the 83 genotypes (73 pre-selected plants from BC5S1, BC4S1, and BC2S1, 2 samples of each parent, 2 additional samples of doubled haploids of the recurrent parent and 2 samples of the F1 hybrid) generated a total of 201,181,531 reads, with a mean of 2,423,873 reads per genotype. After the filtering steps, more than 85% clean reads were aligned to the 12 pseudomolecules of the new eggplant genome developed by the Italian Eggplant Genome Sequencing Consortium (Barchi et al., 2016). The SNP calling allowed identification of a total of 58,401 polymorphisms. Several filters were applied to the VCF file, using NGS_CRUMBS software (https://bioinf.comav.upv.es/ngs_crumbs/), in order to select the most suitable variants for assessment using the HRM technique. Although, all polymorphisms matched the quality criteria, a subset of 796 was selected for the genotyping.

**Figure 3:**
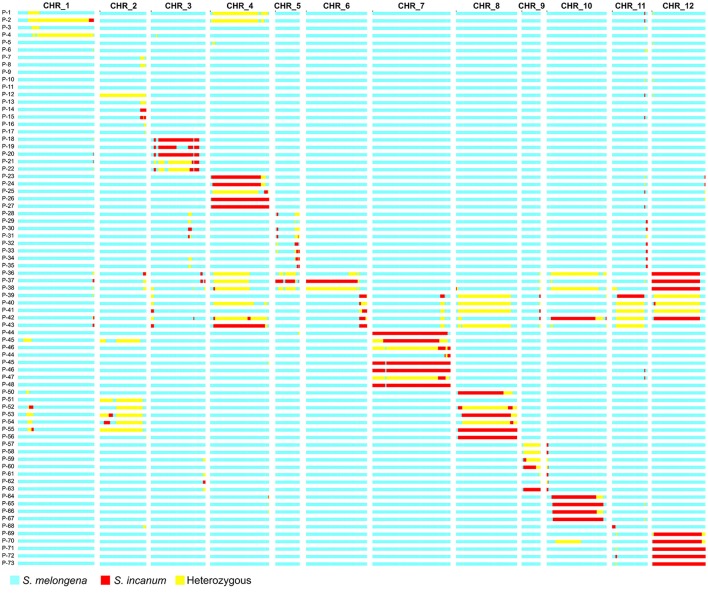
Graphical genotypes of the selected plants genotyped by genotyping-by-sequencing (GBS) after four to five (two for P-36 to P-43) rounds of backcross (typically five, but two or four for some genotypes) and one round of selfing, using *Solanum incanum* MM577 as the donor parent in the genetic background of *S. melongena* AN-S-26. The first row indicates chromosome number and the first column the plant code. Homozygous introgressions of the donor parent are depicted in red, while the genetic background of the recipient parent is depicted in blue and the heterozygous regions in yellow.

The mean number of SNPs per chromosome was 66, with a range of 38 (chromosomes 9 and 11) to 92 (chromosome 3). Using the subset of 796 filtered SNPs, increasing the number of markers for genotyping over elevenfold, more than 90% of introgressions were confirmed by GBS, as well as the segregation of the heterozygous regions previously analyzed by manual genotyping. A total of 36% of the introgressions was totally homozygous for the donor parent, 21.3% were still totally heterozygous, and 33.3% of them displayed a combination of homozygous and heterozygous regions in the first round of selfing (Figure 3).

Double recombinations, which were practically undetected in the previous manual genotyping, were identified in the regions with the highest density of molecular markers. In fact, the distribution of SNPs was very uneven along the chromosomes, being generally greater in the euchromatin and less in the centromeric regions. The development of the backcross generations without screening the genetic background of the selected progenies until the first round of selfing led to the dragging of non-target introgressions. In fact, 84% of the genotyped plants presented at least one non-target introgression, with the most common case being two non-targeted introgressions per plant (37.3%), followed by one (26.6%) and three (8%) non-target introgressions per plant. Obviously, the plants selected for chromosome 6 were those that presented the highest number of non-targeted introgressions (from seven to eleven) due to the fact that they were backcrossed just twice instead of being subjected to four or five rounds of backcrosses of the others.

However, in most cases, the non-target introgressions of the 73 ABs progenitors (i.e., selected BC5S1, BC4S1, and BC2S1 plants) were smaller than 5 Mb and positioned on the edges of the chromosomes, while few others were larger, especially those of chromosome 6, and located in the centromeric regions where generally the recombination is low. Most of the 159 non-target introgressions in the 73 ABs progenitors were heterozygous (60.3%), which meant an easier elimination by segregation through selfing, while 52 non-target introgressions (32.7%) were homozygous for the donor parent, and 11 (7%) were both heterozygous and homozygous in different percentages. The negative selection against the homozygous non-target introgressions is more complicated compared to the heterozygous ones, requiring at least an additional round of backcross before the elimination by segregation in a subsequent selfing generation.

However, genetic characterization by GBS revealed the high coverage of the donor parent across the ABs progenitors (Table 1). In fact, thanks also to the non-target introgressions, the representation of *S. incanum* was around 99% in the AB materials, while the other 1% belonged to non-polymorphic regions between the two parentals. This means that no region of the donor parental had been lost during the backcross process and all the *S. incanum* genome had been introgressed into the ABs, although some materials will need more rounds of backcrossng and selfing to produce ILs with just one introgression in homozygosis.

**Table 1:** Statistics of *Solanum incanum* MM577 genome coverage in the 73 progenitors (corresponding to selected plants of the BC5S1, BC4S1, and BC2S1 generations) of the advanced backcrosses (ABs) set, after the genotyping by genotyping-by-sequencing (GBS).

**Chrom**.	**Chrom. size (Mb)**	***S. incanum* size (Mb)**	***S. incanum* (%)**	**Heter. regions size (Mb)**	**Heterozygous (%)**	***S. melongena* size (Mb)**	***S. melongena* (%)**
1	136	20.8	15.3	109.7	80.7	5.5	4.0
2	83	25.1	30.2	57.9	69.8	–	–
3	97	92.4	95.2	4.6	4.8	–	–
4	105	105	100.0	–	–	–	–
5	43	43	100.0	–	–	–	–
6	108	108	100.0	–	–	–	–
7	142	142	100.0	–	–	–	–
8	109	106.8	97.9	2.2	2.1	–	–
9	43	43	100.0	–	–	–	–
10	106	95.4	90.1	5.6	5.2	5.0	4.7
11	72	72	100.0	–	–	–	–
12	100	100	100.0	–	–	–	–
Total	1144	953.5	83.4	180.0	15.7	10.5	0.9

*Solanum melongena AN-S-26 regions were assigned when no heterozygous or S. incanum introgressions were present, while heterozygous regions were assigned when no donor parent introgressions were present at the same time. All other regions were considered as S. incanum regions*.

**Figure 4:**
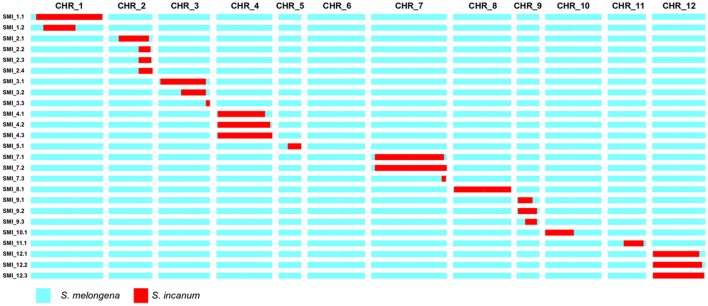
Graphical genotypes of ILs developed using *Solanum incanum* MM577 as the donor parent in the genetic background of *S. melongena* AN-S-26. The first row indicates the chromosome number and the first column the IL code. Homozygous introgressions of the donor parent are depicted in red, while the genetic background of the recipient parent is depicted in blue.

The first round of selfing and the high-throughput genotyping by GBS allowed identification of the first four fixed ILs (SMI_2-1, SMI_7-1, SMI_7-3, SMI_8-1), with two of them (SMI_7-1 and SMI_8-1) covering most of the entire chromosome physical size (Table 2 and Figure 4). Based on the genotype of the target introgressions (homozygous, heterozygous, or both), and the number and the genotype of the non-target introgressions, selected plants of the ABs were selfed or backcrossed in order to obtain new ILs. The progenies obtained were screened with a set of 62 SNPs derived from the GBS and converted for assessment using the HRM technique, the mean marker density being higher in the euchromatin regions and lower in the centromeres. The design of the new markers was based on fixing the single-introgression ILs and reducing the number of non-target introgressions. Hence, the number of SNPs was different for each chromosome and depended on the size of the latter, resulting in four to eight SNPs per chromosome.

**Table 2:** Statistics of the *S. incanum* MM577 introgression lines (ILs) in the genetic background of *S. melongena* AN-S-26 using a 12 pseudomolecule eggplant genome (Italian Eggplant Genome Sequencing Consortium; Barchi et al., 2016), and a number of candidate genes for drought tolerance.

**ILs**	**Chrom.**	**Donor parent (%)**	**IL size (Mb)**	**IL position (Mb)**	**Chrom. IL size (%)**	**Chrom. total ILs size (Mb)**	**Chrom. total ILs size (%)**	**Drought-tolerance related genes^a^**
SMI_1.1	1	9.9	114	19–133	83.8	114	83.8	–
SMI_1.2		0.7	9	27–36	6.5			–
SMI_2.1	2	3.2	37	38–75	44.5	43	51.8	1
SMI_2.2		0.1	2	75–77	2.4			–
SMI_2.3		0.4	5	75–80	6.0			2
SMI_2.4		0.5	6	75–81	7.2			3
SMI_3.1	3	6.9	79	7–86	81.4	82	84.5	7
SMI_3.2		0.6	8	78–86	8.2			2
SMI_3.3		0.2	3	93–96	3.0			1
SMI_4.1	4	7.0	81	4–85	75.2	101	96.1	3
SMI_4.2		8.2	94	4–98	89.5			4
SMI_4.3		8.8	101	4–105	96.1			6
SMI_5.1	5	0.6	8	35–43	18.6	8	18.6	11
SMI_7.1	7	10.5	121	14–135	85.2	125	88.0	4
SMI_7.2		10.9	125	14–139	88.0			7
SMI_7.3		0.8	10	129–139	7.0			4
SMI_8.1	8	9.2	106	3–109	97.2	106	97.2	11
SMI_9.1	9	1.8	21	5–26	48.3	29	64.4	3
SMI_9.2		2.5	29	5–34	64.4			3
SMI_9.3		0.7	9	26–34	20.9			1
SMI_10.1	10	0.1	2	0–2	1.8	2	1.8	–
SMI_11.1	11	0.2	3	60–63	4.1	3	4.1	–
SMI_12.1	12	7.2	83	3–86	83.0	93	93.0	11
SMI_12.2		7.7	89	3–92	89.0			14
SMI_12.3		8.1	93	3–96	93.0			18
Mean		4.3	49.5		48.2	64	62.1	5.8
Total						706		68

*The positions and size of the ILs are represented in megabases (Mb) according to the physical position in each chromosome (Chrom.)*.

a *Orthologous loci related to drought tolerance are reported in the Supplementary Table*.

The genotyping of an additional generation of selfing from the ABs allowed identification of 21 more fixed single-introgression homozygous ILs, raising the total set of ILs to 25 different lines (Table 2). On average, the ILs contained 4.3% of the donor parent genome, ranging from 0.1 to 10.9%, altogether covering 61.7% of the *S. incanum* genome. The mean size of the introgressions was 49.5 Mb (ranging from 2 to 125 Mb), encompassing between 1.8 and 97.2% of the target chromosome length. Chromosome 2 presented the largest number of ILs (4), while chromosome 6 had none. The most covered chromosome was 8, with 97.2% of the total length, and the least covered among those having at least one IL was chromosome 10, with 1.8%.

### Candidate genes for drought tolerance

Due to the lack of genomic and transcriptomic studies in the eggplant genepool, candidate genes from different crops related to drought tolerance were retrieved from the current scientific literature in order to detect the potential genomic region in eggplant and in the recently developed ILs. A total of 142 candidate genes gave a positive Blast hit (cut-off value of 1e-60) in the eggplant genome, most of them involved in the abscisic acid biosynthesis, catabolism and signaling network, in ethylene synthesis and signaling, in osmolyte biosynthesis and cell protection, in aquaporins and detoxification regulation (Supplementary Table). The chromosomes containing most candidate genes for drought tolerance were chromosome 12 with 28 genes, chromosome 5 with 20 genes, and chromosome 10 with 18 genes. The *S. incanum* alleles of these candidate genes were present in the AB materials, while 68 of them were present in the IL set. For the IL set, SMI_12.3 was the line carrying the most candidate genes (18), followed by SMI_12.2 (14 genes), and SMI_5.1, SMI_8.1, and SMI_12.1 (11 genes each) (Table 2).

## Discussion

*S. incanum* is a close relative of eggplant and its hybrids and backcrosses with *S. melongena* are fully fertile (Knapp et al., 2013; Kouassi et al., 2016; Plazas et al., 2016).

The development of the ABs and ILs of *S. incanum* in the genetic background of *S. melongena*, in its early stages, was characterized by a lack of information available for genotyping the progenies efficiently. This led, at the beginning of the process, to the use of markers for low–medium throughput genotyping from information obtained in other eggplant studies and to the generation of new molecular markers more suitable for our materials. Although, in general some molecular markers can be useful and polymorphic across species (Saha et al., 2004; Barbará et al., 2007), their identification and amplification can be very challenging. In fact, the markers used for genotyping the BC1 generation were developed in other eggplant segregating populations (Nunome et al., 2009; Wu et al., 2009), although they were progressively substituted by markers identified in our population. From the initial set of markers, just one fifth were polymorphic in our BC1 population, which led to a low saturation of some chromosomal regions, which reduced the efficiency for selecting the most appropriate genotypes.

Our strategy for this first step was to select 12 BC1 plants, each one with one chromosome completely heterozygous for the markers from the donor, and at the same time with a higher percentage of the recurrent parent background in the other 11 chromosomes. This approach, consisting of the development of a set of chromosome substitution lines, accelerated the process of recovering the recipient parent background using a small set of selected plants, avoiding the tracking many families until the first round of selfing.

Unfortunately, this strategy did not last long, because, during the selection of the BC2 generation, no plants with complete selected chromosomes heterozygous for the markers screened were found in the progeny of 3 out of the 12 BC1 plants. The same drawback occurred also in further selection steps in the BC3 and BC5 generations. In addition, to reduce the time and resources needed during selection, we genotyped the plants only for the target chromosome instead of for the whole set of chromosomes. The strategy was to develop ILs without screening the genetic background, for the sake of time and resource saving, until AB generations or even selfing rounds were performed also by other authors (Eichten et al., 2011; Pea et al., 2013). In fact, no affordable high-throughput genotyping platforms or SNP arrays were available in eggplant to genotype a large number of plants with a wide set of SNPs in a very short time, as in the major crops like maize (Ganal et al., 2011), wheat (Wang et al., 2014), tomato (Sim et al., 2012), and potato (Vos et al., 2015); so, in our case, all the markers were assessed manually using low–medium throughput genotyping. Moreover, more than half of the markers assessed were SSRs (24 COSII and 36 SSRs), which are very robust and reliable (Varshney et al., 2005), but compared to SNPs are generally more difficult to automate and more time-consuming and expensive, due to the need of detection through an agarose or polyacrylamide gel or capillary sequencing (Jones et al., 2007). Some authors, Yang et al. (2011), calculated that assessing SNPs through a high-throughput genotyping platform can be 100-fold faster and 75% less expensive than SSR gel-based or capillary methods.

All these combined circumstances made the genotyping and selection steps the bottlenecks in the AB and IL development process, more than the interspecific crossing of two genetically distant species, which probably discouraged attempts to start sets of ILs with eggplant genepool materials. In addition, in part due to the excessive amount of time needed for the genotyping and selection steps, and also due to difficulties in obtaining seeded fruit set during the winter–spring season (e.g., slow plant growth, poor flowering with reduced production of pollen, low pollen viability, and parthenocarpy), just one cycle of backcrossing per year was feasible. This meant a very long process of 8 years has been required to obtain a first set of ILs.

For other crops, where two or even three growing cycles per year are possible, the entire set of ILs could be obtained in 3 or 4 years (Eduardo et al., 2005; Barrantes et al., 2014). The strategy to bypassing the screening of the non-targeted chormosomes of the selected plants, even if it has the advantage of reducing the time and the cost for genotyping, increases the number of rounds of backcrosses required to recover the almost totality of the recipient parent background (Frisch et al., 1999). In fact, although generally five rounds of backcross are recommended to recover more than 98% of the recurrent parent, due to the fact that the donor genome proportion is halved with each backcross generation, an adequately high percentage of the recipient parent can be recovered after three generations of marker-assisted backcrossing (Tanksley et al., 1989; Hospital et al., 1992; Hospital and Charcosset, 1997), although this depends on the number of plants screened and the number of markers adequately distributed along the genome (Frisch and Melchinger, 2005).

High-throughput genotyping in the first generations of backcrossing not only accelerates the process of obtaining ILs, but also provides early detection of non-target introgressions and easy elimination of them during the backcross process. When the genetic background is not screened until advanced generations of backcrossing or even after the final collection of ILs, the presence of non-target introgressions is almost certain, as happened in several studies (Shen et al., 2001; Eichten et al., 2011; Pea et al., 2013; Arbelaez et al., 2015). However, non-target introgressions can go unnoticed, until a massive genotyping is performed, even when a low-density genotyping is performed during the IL development process, due to double recombination events undetected by two adjacent markers (Xu et al., 2010; Schmalenbach et al., 2011; Zhang et al., 2011).

Our case was similar to the first scenario. In fact, the genetic background of the progenies was not screened until the first round of selfing when a GBS was performed. The majority of the plants genotyped contained at least one non-target introgression (84%), although most of them were small and regularly placed in certain regions, which mostly corresponded with low-recombination regions. This finding allowed the characterization of the ABs, but caused indirectly a further delay in the development of a set of nearly isogenic lines due to the necessity to clean the genetic background of the non-target introgressions.

Given the high representation of the donor genome in the AB materials, the removal of non-target introgressions will allow obtainment of a complete set of single-introgression fixed ILs where more than 95% of the donor genome is represented. Also, double recombinants were identified by GBS both in target and non-target introgressions. The medium-density genotyping applied during the population development did not allow detect of all the double-crossing-over events, mainly in the euchromatin regions where the recombination rate is higher (Wang et al., 2006; Gaut et al., 2007). In fact, although the selection of the markers before GBS was based on the genetic map, which tends to represent more the euchromatic regions and less the centromeric regions (Zhang W. et al., 2014), some high-recombination regions were not properly saturated due to the fact that the map was already under development. Then, after the GBS, for a more efficient genotyping design, the selection of the new SNPs was based on a combination of the genetic and physical position, plus the necessary markers to screen the non-target introgressions.

Selection based just on genetic maps could under-represent telomeric regions and drag large centromeric regions (Wang et al., 2000), as happened in our case, while a selection based exclusively on physical maps tends to reduce the adequate marker density of high-recombination regions, running the risk of not detecting double recombinants and of over-representing centromeric regions (Barrantes et al., 2014), which is not cost-effective due to the low-recombination ratio. The most efficient approach for reducing the time for the IL population development, avoiding most of the non-target introgressions and double recombinants, and detecting regions undetected by low–medium genotyping, could be to perform high-throughput genotyping in the first cycles of backcrossing. In this way, most of the ILs can be developed just in four generations, as was the case for Barrantes et al. (2014), who developed a set of tomato ILs with *Solanum pimpinellifolium* introgressions, where most of the lines derived from a BC3S1 generation. Even though a few years ago the cost of massive genotyping techniques was high and prohibitive for many labs, currently, the decreasing cost of sequencing and the emergence of new genotyping technologies, like GBS and similar (Campbell et al., 2015), have made the task of genotyping more affordable and precise. This makes possible the development of experimental populations for non-model crops for which genomic resources are limited (Sallam et al., 2015).

The ABs and ILs developed may be of great interest not only for dissecting the genetic basis of domestication traits, but also for developing varieties of eggplant with new traits, including complex ones like drought resistance, introgressed from *S. incanum*. Drought is considered by many experts the stress that presents the strongest negative impact on yield and crop productivity (Krannich et al., 2015). Plants have developed different strategies, involving several different genes and pathways, to mitigate the negative effects of drought (Reguera et al., 2012). In this respect, *S. incanum*, being distributed from northern Africa to the Middle East, is able to survive in desert and dryland areas (Ranil et al., 2015; Vorontsova and Knapp, 2016) and, therefore, is a potential source of variation for tolerance to drought, as demonstrated by Savarino (2017). However, almost nothing is known about the mechanisms and strategies used by *S. incanum* to tolerate this abiotic stress. For the major crops, like rice and wheat (Tang et al., 2016; Valluru et al., 2016), several mechanisms have been described involving many genes and pathways required for tolerance to drought. In this study, we have identified 142 eggplant genome regions that are orthologs of candidate genes related to drought tolerance in other crops; while all these genome regions are represented in the AB materials, 68 of these regions are present in the ILs newly developed using a donor parent (*S. incanum* MM577) that is much more resistant to drought than the recipient parent (*S. melongena* AN-S-26) (Savarino, 2017). Some of these ILs are very promising, carrying several orthologs related to drought tolerance from different crops. Monitoring the expression of these regions during a screening test for drought tolerance using the AB materials and the subset of ILs could pave the way for understanding the regulatory mechanisms for drought tolerance in eggplant. At the same time, it could facilitate the development of new eggplant varieties in a scenario when drought and desertification are increasingly a serious constrain for crop production.

A preliminary drought screening test realized by Savarino (2017), using intermediate backcross materials during the IL development, showed significant drought-tolerance levels among the parents, hybrid and the backcross materials. Although, the results were promising, the screening was exploratory and a further analysis is required. In addition, Sotomayor (2016) realized a phenotypic characterization of 62 AB materials for morphological traits related to fruit, flower, prickles, and chlorogenic acid content. A great variation among the materials was found for almost all the traits (Supplementary Image). Once again, these results are preliminary and more extensive analyses are being undertaken to discover QTLs and dissect the genetic basis of important agronomic traits. In the meantime, and without any further delay, the collection of ABs and ILs is available for the scientific community.

In conclusion, in this study, using the available genomic information (reduced at the beginning and more comprehensive at the later stages), we have developed the first set of ABs and ILs in eggplant that cover the whole genome of a wild donor species (*S. incanum*). These materials will have a tremendous impact in a wide range of studies in this genepool, which was lagging behind compared to other important crops of the same genus, like tomato and potato. In fact, precision QTL mapping in the ABs and ILs will allow confirmation of existing QTLs (Buerstmayr et al., 2011; Sayed et al., 2012) and detection of new ones, which has been complicated until now by the lack of an experimental population for this crop. In addition, new genetic diversity has been introduced in a crop that had suffered a reduction in the genetic base due to repeated genetic bottlenecks during the domestication process (Muñoz-Falcón et al., 2009; Meyer et al., 2012). The ABs and ILs developed could be used directly by breeders in their breeding pipelines, as most of them do not display undesirable wild traits because they just have a small percentage of the donor parent genome. We have provide further evidence that the implementation of high-throughput genotyping technologies can have an extraordinary impact on the development and precision of IL development, reducing the time and, probably, the cost of obtaining this segregating population (Schmalenbach et al., 2011; Arbelaez et al., 2015). Also, the ABs and ILs developed up to now will allow isolation of improved versions of the IL population, which will further contribute to the enhancement of eggplant breeding.

## Author contributions

JP and SV planned the study. PG, JP, and SV supervised the research. PG, GM, MP, and FH generated the ABs and ILs collection. PG managed the seed collections. PG, GM, and MP performed the genotyping. FH contributed to the GBS analysis. PG, JP, and SV drafted the manuscript. All authors read and approved the final manuscript.

### Conflict of interest statement

The authors declare that the research was conducted in the absence of any commercial or financial relationships that could be construed as a potential conflict of interest.

## Abbreviations

AB: advanced backcross
COSII: conserved ortholog set of genes II
HRM: high resolution melting
IL: introgression line
QTL: quantitative trait locus
RIL: recombinant inbred line
SNP: single nucleotide polymorphism
SSR: simple sequence repeat
UPV: Universitat Politècnica de València.
