# Supplementary figures and images for: Development and Genetic Characterization of Advanced Backcross Materials and An Introgression Line Population of Solanum incanum in a S. melongena Background
Source: Front Plant Sci. 2017 Aug 30;8:1477. doi: 10.3389/fpls.2017.01477 (PMC5582342; doi:10.3389/fpls.2017.01477)

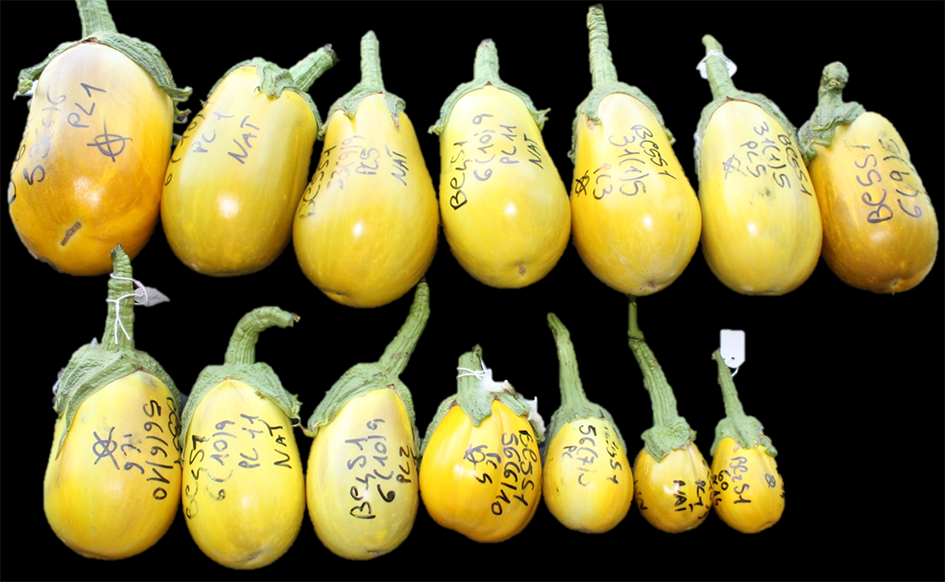

Supplement: Supplementary file 2 [file Image1.TIF]
